# Supplementary material for: Energetic and physical limitations on the breaching performance of large whales
Source: eLife. 2020 Mar 11;9:e51760. doi: 10.7554/eLife.51760 (PMC7065846; doi:10.7554/eLife.51760)
Supplement: Supplementary file 1. — (A) Lower and upper bounds of daily Field Metabolic Rate (FMRdaily) for five humpback whales across a range of sizes. FMRdaily, WM was calculated using the equation for marine mammal FMRdaily proposed by Williams and Maresh (2015). FMRdaily, Nagy was calculated using the equation for terrestrial mammal FMRdaily proposed by Nagy (2005) and multiplied by 1.5. The cost of a high-performance breach and a single high-performance lunge are expressed as percentage of daily energy budget. B) Kinematic and morphological parameters used to calculate the energetics of breaching and lunge feeding. [file elife-51760-supp1.docx]

| length  (m) | mass  (kg) | *FMR*_daily, WM_  (MJ/day) | cost  (% of *FMR*_daily, WM_) | | *FMR*_daily, Nagy_  (MJ/day) | cost  (% of *FMR*_daily, Nagy_) | |
| --- | --- | --- | --- | --- | --- | --- | --- |
|  |  |  | breach | lunge |  | breach | lunge |
| 7.8 | 7000 | 190 | 0.5 | 0.4 | 1100 | 0.08 | 0.06 |
| 10.5 | 17000 | 280 | 1.0 | 0.4 | 2300 | 0.12 | 0.05 |
| 12.7 | 30000 | 360 | 1.0 | 0.4 | 3700 | 0.10 | 0.04 |
| 14.7 | 46000 | 440 | 2.2 | 0.6 | 5200 | 0.19 | 0.05 |
| 14.8 | 46000 | 440 | 2.3 | 0.8 | 5200 | 0.20 | 0.07 |

**Table S1A. Lower and upper bounds of daily Field Metabolic Rate (*FMR*_daily_) for five humpback whales across a range of sizes.** *FMR*_daily, WM_ was calculated using the equation for marine mammal *FMR*_daily_ proposed by Williams and Maresh (2015). *FMR*_daily, Nagy_ was calculated using the equation for terrestrial mammal *FMR*_daily_ proposed by Nagy (2005) and multiplied by 1.5. The cost of a high-performance breach and a single high-performance lunge are expressed as percentage of daily energy budget.

| species | length (m) | width (m) | mass (kg) | *T_acc_* (s) | | *T_plat_* (s) | | *U_i_* (m/s) | | *U_f_* (m/s) | | *a_avg, acc_* (m/s^2^) | |
| --- | --- | --- | --- | --- | --- | --- | --- | --- | --- | --- | --- | --- | --- |
|  |  |  |  | breach | lunge | breach | lunge | breach | lunge | breach | lunge | breach | lunge |
| humpback | 7.8 | 1.4 | 7000 | 8.0 | 4.7 | 0.0 | 2.1 | 1.6 | 1.8 | 6.2 | 5.3 | 0.6 | 0.7 |
| humpback | 10.5 | 2.3 | 17000 | 8.1 | 4.7 | 0.0 | 0.0 | 1.6 | 1.8 | 7.1 | 5.0 | 0.7 | 0.7 |
| humpback | 12.7 | 2.3 | 30000 | 5.6 | 2.9 | 3.5 | 0.0 | 1.8 | 2.8 | 6.0 | 5.0 | 0.8 | 0.8 |
| humpback | 14.7 | 2.7 | 46000 | 8.5 | 3.3 | 0.0 | 0.0 | 1.5 | 2.0 | 8.2 | 4.8 | 0.8 | 0.9 |
| humpback | 14.8 | 2.8 | 46000 | 12.7 | 6.1 | 0.0 | 0.0 | 1.8 | 1.8 | 8.1 | 5.4 | 0.5 | 0.6 |
| blue | 25.2 | 2.9 | 104000 | - | 9.7 | - | 0.0 | - | 1.7 | - | 5.7 | - | 0.4 |

**Table S1B. Kinematic and morphological parameters used to calculate the energetics of breaching and lunge feeding.**
